# Safety, tolerability, and pharmacokinetics of anti-SARS-CoV-2 monoclonal antibody SA55 injection in healthy participants

**DOI:** 10.1128/aac.00568-25

**Published:** 2025-07-17

**Authors:** Yibo Zhou, Xing Meng, Jianhua Li, Gang Zeng, Jin Wang, Yunlong Cao, Chaoying Hu, Ronghua Jin

**Affiliations:** 1 Phase I Clinical Research Center, Beijing Ditan Hospital, Capital Medical University 12517, Beijing, China; 2 Public Health Emergency Management Innovation Center, Beijing, China; 3 Clinical Research and Development Center Sinovac Biotech Co., Ltd., Beijing, China; 4 Zhejiang Key Laboratory of Public Health Detection and Pathogenesis Research, Zhejiang Provincial Center for Disease Control and Prevention 117838, Hangzhou, China; 5 Biomedical Pioneering Innovation Center (BIOPIC), Peking University 12465, Beijing, China; 6 Changping Laboratory 662243 , Beijing, Beijing, China; IrsiCaixa Institut de Recerca de la Sida, Barcelona, Spain

**Keywords:** SARS-CoV-2, monoclonal antibody, SA55 injection, pharmacokinetics

## Abstract

This study aimed to evaluate the safety, tolerability, pharmacokinetics (PK), immunogenicity, and serum neutralizing activity of a single dose of SA55 injection in healthy individuals. A randomized, controlled, double-blind trial was conducted with 40 healthy volunteers aged 18–65 years. Participants received a single intramuscular injection of the investigational product at four dose levels (150 mg, 300 mg, 600 mg, and 900 mg), with randomization stratified by dose cohort. Dose proportionality and linearity of PK parameters (AUC_0-∞_, AUC_0-t_, and C_max_) were assessed using one-way ANOVA and the Power model, while neutralizing the activity against the EG.5 strain, and safety outcomes were monitored. Results demonstrated that SA55 injection was safe and well-tolerated at doses ranging from 150 mg to 900 mg, with a half-life of 94–103 days and no significant dose-dependent trends. C_max_, AUC_0-t_, and AUC_0-∞_ showed an approximately linear relationship with dosage, while T_max_ decreased as the dosage increased. The levels of ADA of all positive participants were at low levels, and the cumulative positive rates among treatment groups were similar to that of the placebo group. The neutralizing activity peaked at 3–4 days post-administration, with the 600 mg dose exhibiting comparable activity to the 900 mg dose and superior activity to lower doses, suggesting it as a potential target dose. In conclusion, SA55 injection demonstrated excellent safety, tolerability, and neutralizing activity against SARS-CoV-2 in healthy populations, with the 600 mg dose emerging as a promising candidate for further development.

**CLINICAL TRIALS:**

This study is registered with ClinicalTrials.gov as NCT06050460.

## INTRODUCTION

Starting from late 2019, a novel acute respiratory disease caused by SARS-CoV-2 spread rapidly worldwide. The WHO declared it a Public Health Emergency of International Concern (PHEIC) (1) on 31 January 2020 and later classified it as a pandemic (2). The virus was officially named “Severe Acute Respiratory Syndrome Coronavirus 2 (SARS-CoV-2)” by the International Committee on Taxonomy of Viruses (ICTV), while the WHO designated the disease as COVID-19 on 12 February 2020 (3).

During the COVID-19 pandemic, various vaccines and therapies for COVID-19 were widely implemented, making significant contributions to the prevention and control of the disease (4, 5). The continuous evolution and emergence of SARS-CoV-2 variants pose significant challenges to controlling the global pandemic and raise concerns about the efficacy of monoclonal antibody therapies and vaccines (6, 7). Neutralizing antibody (NAb) drugs against SARS-CoV-2 have shown good efficacy in preventing or treating COVID-19 by directly binding to the virus, thereby inhibiting further infection. According to data published by COVID-NMA, as of 16 October 2022, there are more than 370 investigational drugs for the novel coronavirus globally (8). The U.S. Food and Drug Administration (FDA) has authorized the emergency use of several SARS-CoV-2 antibody therapies during the pandemic, including monotherapies such as tocilizumab (Roche), 1% propofol-lipuro injection (B. Braun), bamlanivimab (Lilly) (9), and sotrovimab (GSK) (10); as well as combination therapies like Evusheld (tixagevimab/cilgavimab) (AstraZeneca) (11), REGEN-COV (Regeneron) (12, 13), and bamlanivimab/etesevimab (Lilly) (14, 15). There are currently over 80 candidate drugs for COVID-19 in China approved to initiate clinical trials, with 12 candidate drugs having entered the clinical stage. As far as we know, the combination therapy of bimagrumab and romlusevimab by Brii Biosciences was conditionally approved for marketing by the National Medical Products Administration (NMPA) in December 2021 (16).

With the continuous mutation of the virus, many of the currently available neutralizing antibodies do not meet the needs for COVID-19 prevention and treatment (17). Due to the rapid mutation of the spike protein of the novel coronavirus and since neutralizing antibodies are designed to target the structure of the virus’s spike protein on cells, the use of neutralizing antibodies is limited by the sensitivity of circulating strains to these antibodies (18). This has led to the emergence of many variants, posing new challenges for COVID-19 (7, 19–23). With the global prevalence of the Omicron variant, the *in vitro* activity of various neutralizing antibodies against the Omicron strain has rapidly diminished (7). New variants have the ability to evade antibodies obtained through vaccination or previous SARS-CoV-2 infections, as well as neutralizing antibody (NAb) drugs targeting SARS-CoV-2 (24). Currently, the monoclonal antibody treatments for COVID-19 approved for Emergency Use Authorization (EUA) target the receptor-binding domain (RBD) of the SARS-CoV-2 spike protein. Although monoclonal antibody combinations (antibody cocktail therapies (25–27) have been developed to prevent potential neutralization escape by targeting multiple viral epitopes, many neutralizing antibody treatments are still evaded by variants of the novel coronavirus (20, 21, 23, 28). Currently, the FDA has revoked all Emergency Use Authorizations for the previously issued COVID-19-neutralizing antibodies. Therefore, developing broad-spectrum neutralizing antibodies has become one of the crucial strategies for the global response to the rapid mutation of the novel coronavirus.

The ideal drug for treatment should have characteristics of rapid onset and strong potency, as well as broad-spectrum efficacy to address various variants of the novel coronavirus. To achieve these goals, a novel broad-spectrum neutralizing antibody injection, anti-SARS-CoV-2 monoclonal antibody SA55 injection (SA55 injection), was developed. The SA55 injection can bind to the novel coronavirus, thereby preventing the virus from entering host cells and blocking infection. The SA55 antibody was selected using an innovative high-throughput sequencing technology from a library of approximately 13,000 antibodies, which efficiently binds to conserved sites shared by various coronaviruses that are less prone to mutation (24, 29). It effectively neutralized multiple Omicron variants *in vitro*, including BA.1, BA.2, BA.4/5, BF.7, XBB, BQ.1, and BQ.1.1 (30–35).

In this Phase I clinical trial, we aimed to evaluate the safety and tolerability of a single intramuscular injection of SA55 injection in healthy populations, as well as its PK characteristics, immunogenicity, and serum neutralizing activity post-administration.

## MATERIALS AND METHODS

### Study drugs

This study employs a placebo-controlled design, with both the investigational drug and placebo developed and produced by Sinovac Life Sciences Co., Ltd. (Sinovac). The anti-SARS-CoV-2 monoclonal antibody SA55 injection (Batch number: KC202301001A, Production date: 3 January 2023); expiry date: 2 January 2025) is composed primarily of the broad-spectrum neutralizing antibody SA55, present at a concentration of 150 milligrams per milliliter. Excipients include hydrochloric acid histidine, hydrochloric acid arginine, histidine, sucrose, and polysorbate 80 (II). The placebo (Batch number: 20221228, Production date: 29 December 2022; expiry date: 28 December 2022) does not contain neutralizing antibody SA55. Its components include hydrochloric acid histidine, hydrochloric acid arginine, histidine, sucrose, polysorbate 80 (II), and water for injection.

### Study participants

Each healthy volunteer gave written informed consent to participate in this study after being told of the objectives, procedures, and possible risks of the research. The study was conducted in accordance with guidelines of the Declaration of Helsinki, Good Clinical Practices, and relevant regulatory standards. The researchers screened the volunteers, including vital signs, physical examinations, 12-lead electrocardiography, and laboratory tests (complete blood count, urinalysis, hematology, blood biochemistry, coagulation function, urinalysis, drug abuse screening, and hepatitis/HIV testing). And in the judgment of the study physician, the subjects did not have any other factors that made them unsuitable for clinical study.

Main inclusion criteria were as follows: healthy males or females, aged 18 to 65 years; male volunteers weighed ≥50.0 kg; female volunteers weighed ≥45.0 kg, with a body mass index (BMI) between 18.0 and 28.0 kg/m²; had no plans for childbearing or sperm/egg donation during the study period, and voluntarily adopted effective contraceptive measures. Main exclusion criteria include the following: with known allergies to the investigational drug or any components of the formulation or to other similar drugs; poorly controlled chronic diseases or a history of severe illnesses; potential interference at the target injection site (deltoid muscle of the upper arm) that may affect drug administration or local reaction observation; received any SARS-CoV-2 neutralizing antibody injection prior to screening; with known history of SARS-CoV-2 infection or vaccination against COVID-19 within the past 3 months.

### Study design

**Fig 1:**
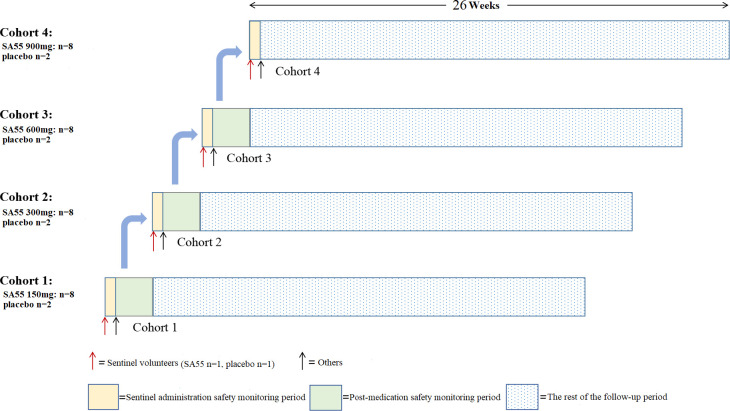
Study design flowchart.

This study employs a randomized, controlled, double-blind design, recruiting a total of 40 healthy volunteers aged 18 to 65 years. Participants were divided into four dosage groups, with 10 for each. After providing informed consent, volunteers were screened against the inclusion/exclusion criteria and sequentially enrolled from the lowest- to the highest-dose group, as illustrated in Fig. 1, and received either 150 mg, 300 mg, 600 mg, or 900 mg of the investigational drug or placebo. Participants of cohort 1 (150 mg dose group) were enrolled first. The enrollment of the next cohort (higher dose group) would only be initiated after at least 7 days of safety follow-up for the lower-dose group after administration (including adverse events (AEs), physical examinations, vital signs, 12-lead electrocardiogram tests, complete blood count, urinalysis, blood biochemistry, and coagulation function tests), and at least confirmation of no significant safety concerns.

Within each dosage group, participants were randomly assigned in a 4:1 ratio to receive either SA55 injection or an equivalent volume of placebo (with eight participants receiving SA55 injection and two receiving placebo). Each dosage group had a “sentinel dosing” setup. Specifically, on the day of dosing, two participants were first enrolled and randomly assigned in a 1:1 ratio to receive either SA55 injection or placebo. After monitoring for at least 24 hours and confirming no safety concerns, the remaining eight participants (with seven receiving SA55 injection and one receiving placebo) were then randomly assigned to the group. All participants had blood samples collected at various time points during the study to measure drug concentrations, anti-drug antibody (ADA) levels, and neutralizing antibody levels, enabling PK analysis, immunogenicity assessment, and neutralizing activity assessment.

### Safety assessment

The safety assessment included monitoring changes in physical examination findings, laboratory tests, and AEs. Vital signs were measured before administration and at 1, 2, 24, 48, 96, 120, 144, and 168 hours post-administration, including blood pressure, pulse, respiration, and temperature. Physical examinations, 12-lead electrocardiography, and laboratory tests (complete blood count, urinalysis, hematology, blood biochemistry, coagulation function, urinalysis, drug abuse screening, and blood/urine pregnancy tests) were performed on days 4, 8, 15, 29, 57, 85, 113, 141, 169, and 183 following administration. AEs were collected during the study through a combination of investigator-directed observations and subject spontaneous reporting and graded according to the CTCAE 5.0.

### Outcomes

The primary objective was to assess the safety and tolerability of the SA55 injection in the healthy population, by evaluating the incidence of AEs (including clinical symptoms, abnormal vital signs, laboratory testing abnormalities, and 12-lead electrocardiogram abnormalities) and SAEs. The secondary objectives included the evaluation of PK characteristics, immunogenicity, and neutralizing activity of a single intramuscular injection of SA55 injection. The serum PK parameters included T_max_, C_max_, elimination half-life (t_1/2_), clearance rate (CL), apparent volume of distribution (Vd), and area under the drug concentration-time curve (AUC_0-t_ and AUC_0-∞_). Levels of serum ADA at different time points were assessed to evaluate the immunogenicity of SA55 injection. The serum neutralizing activity of SA55 against SARS-CoV-2 was assessed at various time points post-administration as well.

### PK assay, ADA assay, and neutralizing activity determination

Blood samples for PK analyses were collected at predose and post dose at 24, 48, 72, 120, and 168 h and Days 12, 15, 22, 29, 57, 85, 113, 141, 169, and 183 for single ascending doses of SA55 injection. The serum concentrations of SA55 were measured using liquid chromatography-tandem mass spectrometry (LC-MS/MS). This method uses SA55-IS as the internal standard compound (IS). The analyte SA55 IS is extracted from the serum sample by trypsin enzymatic hydrolysis. After extraction, it is analyzed by the LC-MS/MS system. The analyte SA55 and the internal standard SA55-IS are monitored by the positive ion electrospray ionization (ESI) mode. This method is specific for the determination of the analyte SA55 and the internal standard SA55-IS in human serum samples, and the standard curve is linear within the range of 1.00–300 µg/mL.

Blood samples for ADAs were collected at predose and Days 22, 29, 57, 85, 113, 141, 169, and 183 post-dose for single ascending doses of SA55 injection. ADAs were qualitatively determined by the ECLIA method based on the affinity capture elution (ACE) technology. Briefly, the serum was acid-hydrolyzed to form a complex with the coated drug. After secondary acid-hydrolyzation, it was transferred to the MSD plate. After incubation with the ruthenium-labeled detection reagent, the signal value was determined by electrochemiluminescence (positively correlated with the ADA concentration). The positive control was prepared with 100% normal healthy human serum (PNHS) to verify the specificity and sensitivity of the method.

Blood samples for neutralizing activity determination analyses were analyzed at predose and postdose at 24, 48, 72, 120, and 168 h and Days 12, 15, 22, 29, 57, 85, 113, 141, 169, and 183 for single ascending doses of SA55 injection. The neutralizing activity determination was measured using the Virus Neutralization Test (VNT) as it is performed in 96-well plates and determines the neutralizing antibody titer based on the cytopathic effect (CPE). In a nutshell, serum samples underwent twofold serial dilutions (4- to 8,192-fold) in duplicate and then were mixed with 100 TCID_50_ virus. After 2 h incubation (37°C, 5% CO₂), Vero cells (1.5 × 10⁴/well) were added and cultured for 120 h. The neutralizing antibody titer was defined as the highest serum dilution showing 50% CPE inhibition. Assay validity was confirmed by the following: (i) virus back-titration (32-320 TCID50/50 µL) and (ii) appropriate control responses (cell, positive/negative controls).

### Statistical analysis

Continuous data were shown as the means ± standard deviation (SD), and a one-way analysis of variance (ANOVA) was used to evaluate the dose proportionality of the PK parameters AUC_0-∞_, AUC_0-t_, and C_max_. The Power model was employed to assess the dose linearity of the PK parameters AUC_0-∞_, AUC_0-t_, and C_max_, and a linear relationship plot of the logarithmic transformed values of the PK parameters AUC_0-∞_, AUC_0-t_, and C_max_ against ln(dose) was created. Statistical descriptions of the neutralizing antibody levels and ADA at each time point after dosing were conducted for each group, based on sample size, geometric mean, and two-sided 95% confidence intervals, with the Clopper-Pearson method used to calculate the 95% confidence intervals. All statistical analyses were performed using statistical software SAS 9.4 (SAS Institute, Cary, North Carolina) or later versions.

## RESULTS

### Characteristics of study participants

**Fig 2:**
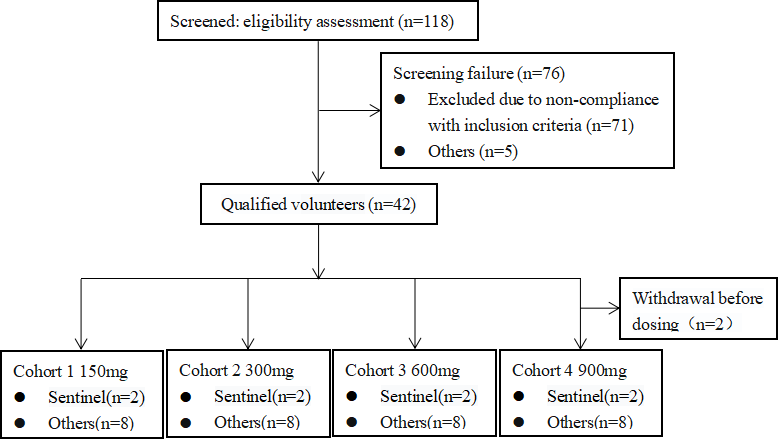
Subject screening flowchart.

As shown in Fig. 2, 40 participants were enrolled and received the investigational drug between 15 June 2023 and 25 January 2024. The demographic characteristics of the participants are detailed in Table 1. The age, sex, ethnicity, height, weight, and BMI of the participants were evenly distributed across groups. Baseline vital signs among participants in each group were generally balanced as well.

**TABLE 1:** Characteristics of study participants^*a*^

Characteristics	SA55-150 mg (*n* = 8)	SA55-300 mg (*n* = 8)	SA55-600 mg (*n* = 8)	SA55-900 mg (*n* = 8)	Placebo (*n* = 8)	Study (*n* = 32)	Total (*n* = 40)
Age (years)							
Mean (SD)	40.6 (12.0)	34.6 (9.3)	42.8 (10.3)	34.4 (9.8)	39.0 (11.5)	38.1 (10.6)	38.3 (10.6)
Gender							
Male n(%)	6 (75.00)	6 (75.00)	8 (100.00)	6 (75.00)	6 (75.00)	26 (81.25)	32 (80.00)
Female n(%)	2 (25.00)	2 (25.00)	0 (0.00)	2 (25.00)	2 (25.00)	6 (18.75)	8 (20.00)
Ethnicity							
Han n(%)	7 (87.50)	7 (87.50)	8 (100.00)	8 (100.00)	8 (100.00)	30 (93.75)	38 (95.00)
Other n(%)	1 (12.50)	1 (12.50)	0 (0.00)	0 (0.00)	0 (0.00)	2 (6.25)	2 (5.00)
Height(cm) Mean (SD)	164.16 (9.61)	166.65 (10.42)	167.05 (5.13)	165.73 (9.75)	162.79 (5.56)	165.90 (8.60)	165.28 (8.12)
Weight(kg) Mean (SD)	63.994 (9.726)	67.625 (10.832)	63.138 (7.032)	63.950 (8.439)	62.475 (7.323)	64.677 (8.844)	64.236 (8.520)
BMI(kg/m^2^) Mean (SD)	23.64 (1.92)	24.33 (2.95)	22.60 (2.22)	23.21 (1.63)	23.59 (2.67)	23.44 (2.22)	23.47 (2.28)

^*a*^ BMI: body mass index. BMI = weight (kg)/height^2^ (m^2^); SD:standard deviation.

### Safety assessment

Throughout the study, all AEs were closely monitored and observed. A total of 40 participants received different doses of SA55 injection or placebo. During the safety observation period, the overall incidence of AEs was 82.50% (33/40), with incidence rates of 87.50% (7/8), 100% (8/8), 87.50% (7/8), 75.00% (6/8), and 62.50% (5/8) for the 150 mg, 300 mg, 600 mg, 900 mg, and placebo groups, respectively. The overall incidence of AEs related to investigational drug (AR) was 22.50% (9/40), with AR incidence rates of 12.50% (1/8), 12.50%, 25.00% (2/8), 37.50% (3/8), and 25.00% for the 150 mg, 300 mg, 600 mg, 900 mg, and placebo groups, respectively.

The overall AR incidence rate for treatment groups was similar to that of the placebo group (21.88% (7/32) vs 25.00% (2/8)). In terms of AR incidence rates among treatment groups receiving different doses of SA55 injection, the AR incidence rates of the 600 mg and 900 mg groups were slightly higher than those of the 150 mg and 300 mg groups, but still close to those of the placebo group. Furthermore, all ARs were transient laboratory abnormalities without related signs or symptoms and were all classified as Grade 1. No SAEs or Grade 3 or higher AEs occurred during the study period (Table 2).

**TABLE 2:** Incidence of adverse reactions during the study period^*a*^

AE terms n(%)	SA55-150 mg (*n* = 8)		SA55-300 mg (*n* = 8)		SA55-600 mg (*n* = 8)		SA55-900 mg (*n* = 8)		Placebo (*n* = 8)		Total (*n* = 32)	
AEs	19	7(87.50)	10	8(100.00)	14	7(87.50)	14	6(75.00)	20	5(62.50)	57	28(87.50)
Total AR	1	1(12.50)	1	1(12.50)	3	2(25.00)	3	3(37.50)	8	2(25.00)	8	7(21.88)
ALT increased	1	1(12.50)	0	0(0.00)	2	2(25.00)	1	1(12.50)	4	1(12.50)	4	4(12.50)
NE decreased	0	0(0.00)	1	1(12.50)	0	0(0.00)	0	0(0.00)	0	0(0.00)	1	1(3.13)
AST increased	0	0(0.00)	0	0(0.00)	1	1(12.50)	0	0(0.00)	0	0(0.00)	1	1(3.13)
Urinary leukocytes (+)	0	0(0.00)	0	0(0.00)	0	0(0.00)	1	1(12.50)	0	0(0.00)	1	1(3.13)
Blood bilirubin increased	0	0(0.00)	0	0(0.00)	0	0(0.00)	1	1(12.50)	0	0(0.00)	1	1(3.13)
GGT increased	0	0(0.00)	0	0(0.00)	0	0(0.00)	0	0(0.00)	3	2(25.00)	0	0(0.00)
Urinary red blood cells (+)	0	0(0.00)	0	0(0.00)	0	0(0.00)	0	0(0.00)	1	1(12.50)	0	0(0.00)

^*a*^ AE: adverse events; AR: adverse events related to the study drug; ALT: alanine aminotransferase; NE: neutrophil; AST: aspartate aminotransferase; GGT: gamma-glutamyltransferase.

### Pharmacokinetic properties

**Fig 3:**
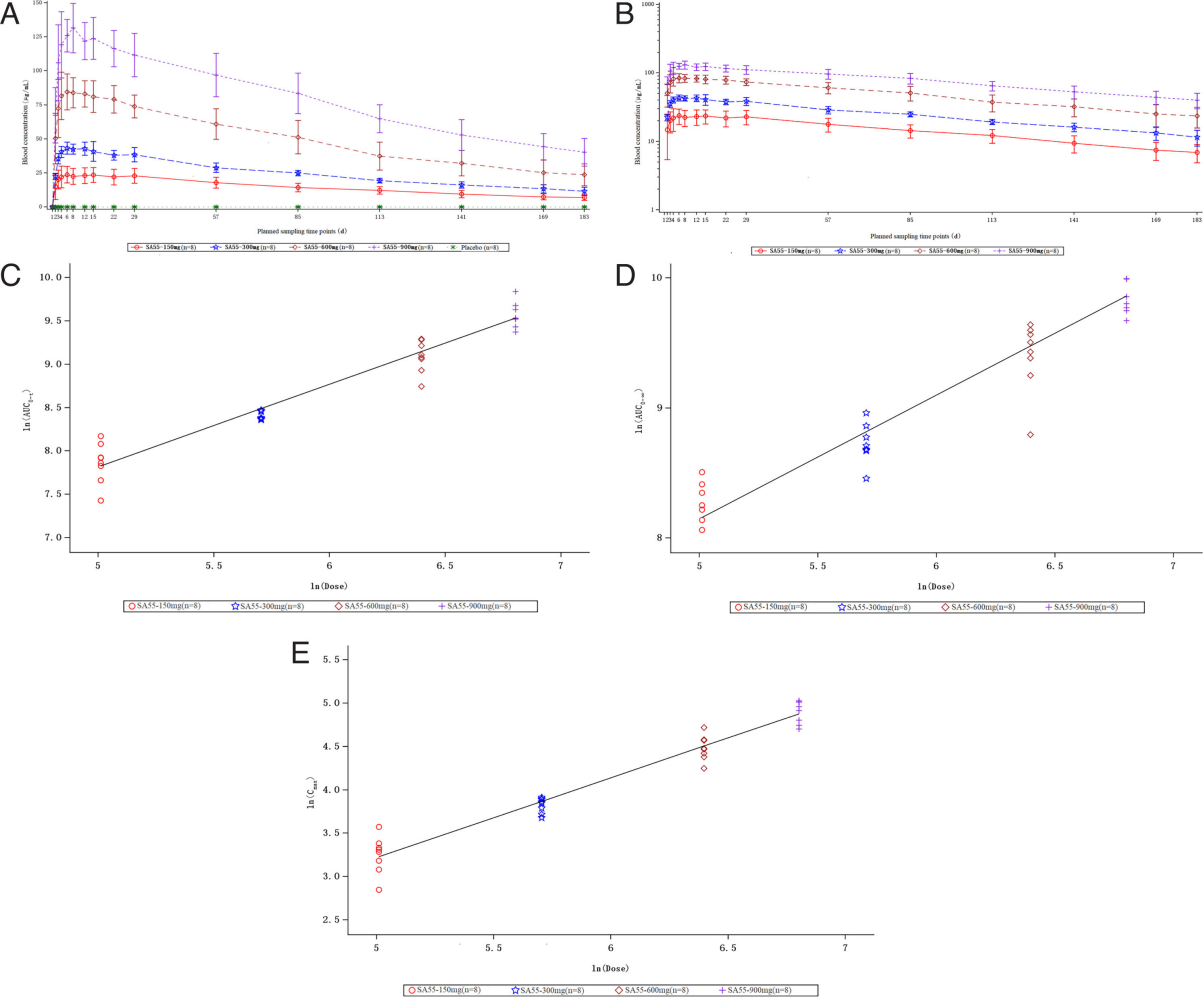
Pharmacokinetics characteristics (PKCS).(**A, B)**：Average drug concentration-time plot after administration from 150 mg to 900 mg for four dose groups (**A**: linear scale, **B**: semi-logarithmic scale). The line plot shows the exposure characteristics of SA55 including AUC_0-t _(**C**), AUC_0-∞ _(**D**), and C_max _(**E**). The error bars denote the SDs.

All participants in the treatment group completed the trial (32 participants, eight in each group) and were included in PK analysis. The blood concentrations of SA55 injection were analyzed at various time points after administration for each dosage group. The average drug concentration-time plots for the four dosage groups after administration are shown in Fig. 3. The results indicated that the blood concentrations in all dosage groups rapidly increased after administration, with peak values observed on either Day 6 or Day 8 post-administration. The peak concentrations for the 150 mg, 300 mg, 600 mg, and 900 mg groups were 23.75 µg/mL, 43.19 µg/mL, 84.53 µg/mL, and 131.50 µg/mL, respectively. After peak concentration, the blood concentrations in the 150 mg and 300 mg groups remained relatively stable within Day 29 and then decreased slowly with time. By Day 183, the blood concentrations in the four dosage groups had decreased to 6.918 µg/mL, 11.566 µg/mL, 23.633 µg/mL, and 40.213 µg/mL, respectively (Fig. 3A and B).

Table 3 summarizes the main PK parameters of each treatment group. The results of the comparison among the four groups indicated that with the increase in dose, T_max_ decreased while C_max_, AUC_0-t_, and AUC_0-∞_ increased, displaying an approximately linear relationship with dosage (Fig. 3C, D and E). With the exception of a slightly longer t_1/2_ in the 150 mg group, t_1/2_ values among other dosage groups were similar, and the clearance rates across groups were also generally consistent, with no significant dose-related correlation observed. CL increased with dose escalation in the first three groups (150–600 mg), which contrasted with its reduction at the 900 mg dose level. Vd exhibited a biphasic profile: an initial rise in the lower-dose groups (150–300 mg) followed by a decline at higher doses (600–900 mg).

**TABLE 3:** Summary of pharmacokinetic parameters (PKPS)^*a*^

	SA55-150 mg (*n* = 8)	SA55-300 mg (*n* = 8)	SA55-600 mg (*n* = 8)	SA55-900 mg (*n* = 8)
C_max_ (µg/mL)				
Mean (SD)	26.300 (5.439)	45.975 (3.884)	89.275 (12.782)	135.000 (17.171)
CV (%)	20.68	8.45	14.32	12.72
AUC_0-t_ (day*µg/mL)				
Mean (SD)	2,653.112 (583.455)	4,492.342 (215.769)	8,990.921 (1,564.070)	14,406.994 (2,199.630)
CV (%)	21.99	4.80	17.40	15.27
AUC_0-∞_ (day*µg/mL)				
Mean (SD)	3,721.567 (920.574)	6,213.618 (924.773)	12,396.362 (2,843.176)	20,257.702 (4,721.263)
CV (%)	24.74	14.88	22.94	23.31
T_max_ (day)				
Median	12.558	11.030	8.005	6.000
Min, Max	3.00, 21.06	3.00, 28.06	3.00, 14.04	3.00, 11.03
t_1/2_ (day)				
Mean (SD)	103.313 (32.457)	96.452 (30.632)	93.543 (23.963)	97.652 (16.192)
CV (%)	31.42	31.76	25.62	16.58
CL (mL/day)				
Mean (SD)	43.344 (14.695)	49.237 (7.469)	51.699 (16.944)	46.161 (8.679)
CV (%)	33.90	15.17	32.77	18.80
Vd (mL)				
Mean (SD)	6,112.825 (1,612.787)	6,581.695 (1,372.026)	6,520.418 (969.441)	6,356.902 (767.663)
CV (%)	26.38	20.85	14.87	12.08

^*a*^ C_max_: peak concentration; AUC_0-t_: curve from 0 to t; AUC_0-∞_: the curve from 0 to ∞ with extrapolation of the terminal phase; T_max_: peak time; t_1/2_: elimination half-life; SD: standard deviation; CV: coefficient of variation; CL: clearance rate; Vd: apparent volume of distribution.

### Assessment of neutralizing activity against the novel coronavirus

**Fig 4:**
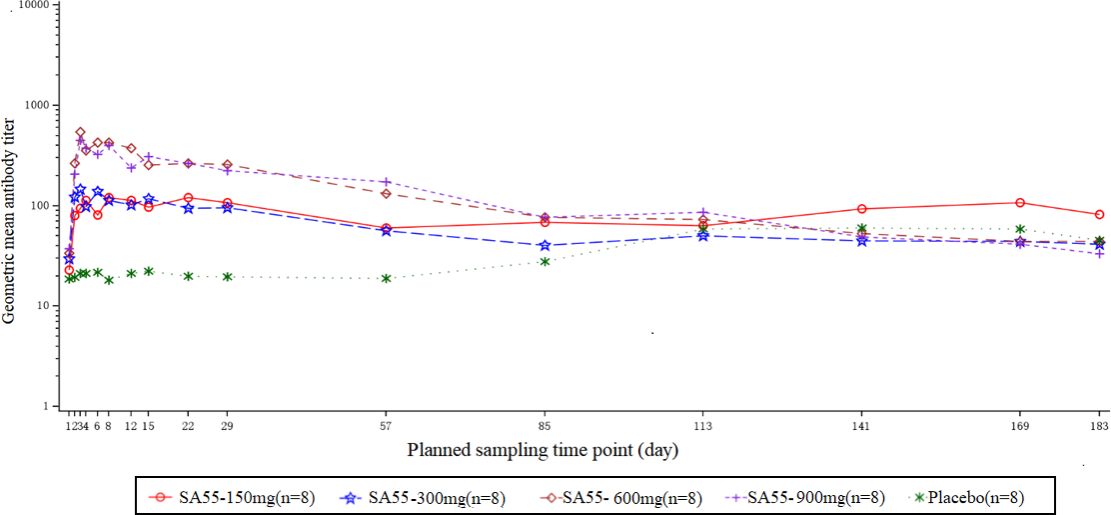
The titer-time semi-log graph of serum neutralizing antibody against SARS-CoV-2 at each time point.

In this study, we assessed the serum neutralizing antibody titers against SARS-CoV-2 (EG.5 strain). The serum neutralizing antibody titers over time for each group are detailed in Fig. 4, presented as a semi-logarithmic plot. Prior to administration (baseline), the neutralizing activities among each treatment group and the placebo group were roughly balanced, with baseline geometric mean titers (GMT) of 23.0, 29.8, 33.7, 37.3, and 18.6 for the 150 mg, 300 mg, 600 mg, 900 mg, and the placebo groups, respectively. After administration, the neutralizing activities significantly increased in all treatment groups. By days 3 to 4 post-administration, the GMTs in each dosage group peaked at 112.5, 145.9, 546.6, and 450.0, with the peak in the 600 mg group significantly higher than that in the 150 mg and 300 mg groups and slightly higher than in the 900 mg group. The serum neutralizing activities in each treatment group gradually declined over time after reaching their peak, with a relatively stable trend in the 150 mg and 300 mg groups before day 29. However, the 150 mg group exhibited a slight increase in the neutralizing activity during the period from day 113 to day 169. By day 183, the levels of neutralizing antibodies in four treatment groups declined to levels similar to those of the placebo group.

### Immunogenicity evaluation

We also conducted immunogenicity assessments for all participants. A total of seven participants tested positive for ADA. The levels of ADA of all positive participants were low (33.60–100.80), and the cumulative positive rates among treatment groups were similar to that of the placebo group (12.5%–25% for treatment groups, 25% for the placebo group) (Tables 4 and 5). Both the levels and incidence of ADA did not demonstrate a direct dose-dependent relationship with the administered dose. Furthermore, no adverse reactions related to the presence of ADA were observed. Overall, the PK and neutralizing activity results suggested that the presence of ADA had no significant impact on the safety, PK characteristics, or neutralizing activity of SA55 injection.

**TABLE 4:** GMT of ADA at each time point^*a*^

GMT	SA55-150 mg (*n* = 8)	SA55-300 mg (*n* = 8)	SA55-600 mg (*n* = 8)	SA55-900 mg (*n* = 8)	Placebo (*n* = 8)
D1 before dosing					
GMT	NA	NA	NA	NA	58.20
OR (95% CI)	NA	NA	NA	NA	0.05, 62,531.68
D22					
GMT	100.80	NA	NA	33.60	33.60
OR (95% CI)	NA	NA	NA	NA	NA
D29					
GMT	100.80	33.60	NA	NA	100.80
OR (95% CI)	NA	NA	NA	NA	NA
D57					
GMT	33.60	33.60	NA	NA	NA
OR (95% CI)	NA	33.60, 33.60	NA	NA	NA
D85					
GMT	33.60	NA	NA	NA	NA
OR (95% CI)	NA	NA	NA	NA	NA
D113					
GMT	NA	33.60	33.60	33.60	58.20
OR (95% CI)	NA	NA	NA	NA	0.05, 62,531.68
D141					
GMT	33.60	NA	33.60	33.60	100.80
OR (95% CI)	NA	NA	NA	NA	0.00, 116,375,317.42
D169					
GMT	NA	NA	33.60	NA	100.80
OR (95% CI)	NA	NA	NA	NA	0.00, 116,375,317.42
D183					
GMT	NA	NA	33.60	NA	33.60
OR (95% CI)	NA	NA	NA	NA	NA

^*a*^ GMT: Geometric Mean Titer. NA: Not applicable. Data are expressed as ORs and 95% CIs. This table analysis was only for volunteers with positive anti-drug antibodies at any point in time after medication.

**TABLE 5:** Cumulative positive rates of ADA at each time point^*a*^

ADA cumulative positive rates	SA55-150 mg (*n* = 8)	SA55-300 mg (*n* = 8)	SA55-600 mg (*n* = 8)	SA55-900 mg (*n* = 8)	Placebo (*n* = 8)
D1 before dosing					
n(%)	0 (0.00)	0 (0.00)	0 (0.00)	0 (0.00)	2 (25.00)
OR (95% CI)	0.00, 36.94	0.00, 36.94	0.00, 36.94	0.00, 36.94	3.19, 65.09
D22					
n(%)	1 (12.50)	0 (0.00)	0 (0.00)	1 (12.50)	1 (12.50)
OR (95% CI)	0.32, 52.65	0.00, 36.94	0.00, 36.94	0.32, 52.65	0.32, 52.65
D29					
n(%)	1 (12.50)	1 (12.50)	0 (0.00)	1 (12.50)	1 (12.50)
OR (95% CI)	0.32, 52.65	0.32, 52.65	0.00, 36.94	0.32, 52.65	0.32, 52.65
D57					
n(%)	1 (12.50)	2 (25.00)	0 (0.00)	1 (12.50)	1 (12.50)
OR (95% CI)	0.32, 52.65	3.19, 65.09	0.00, 36.94	0.32, 52.65	0.32, 52.65
D85					
n(%)	1 (12.50)	2 (25.00)	0 (0.00)	1 (12.50)	1 (12.50)
OR (95% CI)	0.32, 52.65	3.19, 65.09	0.00, 36.94	0.32, 52.65	0.32, 52.65
D113					
n(%)	1 (12.50)	2 (25.00)	1 (12.50)	1 (12.50)	2 (25.00)
OR (95% CI)	0.32, 52.65	3.19, 65.09	0.32, 52.65	0.32, 52.65	3.19, 65.09
D141					
n(%)	1 (12.50)	2 (25.00)	1 (12.50)	1 (12.50)	2 (25.00)
OR (95% CI)	0.32, 52.65	3.19, 65.09	0.32, 52.65	0.32, 52.65	3.19, 65.09
D169					
n(%)	1 (12.50)	2 (25.00)	1 (12.50)	1 (12.50)	2 (25.00)
OR (95% CI)	0.32, 52.65	3.19, 65.09	0.32, 52.65	0.32, 52.65	3.19, 65.09
D183					
n(%)	1 (12.50)	2 (25.00)	1 (12.50)	1 (12.50)	2 (25.00)
OR (95% CI)	0.32, 52.65	3.19, 65.09	0.32, 52.65	0.32, 52.65	3.19, 65.09

^*a*^ ADA: anti-drug antibodies. Data are expressed as ORs and 95% *CIs*. Cumulative positive rates were defined as the presence of at least one positive antibody from before (excluding) dosing to (including) the corresponding time point after dosing.

## DISCUSSION

This study aimed to evaluate the safety, tolerability, serum PK characteristics, immunogenicity, and neutralizing activity of SA55 injection in healthy participants. The study was conducted among healthy adult participants in China, with no significant differences in baseline characteristics among groups. The tolerability among participants was favorable, with no SAEs occurring during the study. The incidence of AR was low following administration of different doses of SA55 and the placebo, and the combined AR incidence rates were similar between placebo and treatment groups (25.00% vs 21.88%). The incidence of ARs in the 600 mg and 900 mg groups was slightly higher than that in the 150 mg and 300 mg groups, yet still comparable to that of the placebo group. The safety evaluation results indicated that a single administration of 150 mg to 900 mg SA55 injection in healthy participants was associated with good safety and tolerability.

In this study, we also assessed the neutralizing activity of serum against the novel coronavirus. We observed that the level of neutralizing antibodies in the 150 mg group increased at the later stage of the study, which was considered to be caused by natural exposure to SARS-CoV-2. Overall, among the dosage groups, the 600 mg and 900 mg groups exhibited better neutralizing activity against the strains compared to the 150 mg and 300 mg groups. However, there was no significant advantage in neutralizing activity when comparing the 900 mg group to the 600 mg group, which may be attributed to the relatively small sample sizes in each group and individual variability among participants. Nonetheless, the results of the serum neutralizing activity suggest that the 600 mg dose maintains good neutralizing activity within 3 months post-administration and may be considered a potential target dose for future studies.

We also observed that in the four trial groups receiving the SA55 injection, the positive rates and cumulative positive rates of participants for ADA at various time points did not significantly change with increasing doses, and all positive participants had low levels of antibody GMT. The levels and incidence of ADA did not demonstrate a direct dose-dependent relationship with the administered dose. Furthermore, no adverse reactions related to anti-drug antibodies were observed in the study. Overall, the PK and neutralizing activity results suggest that the presence of anti-drug antibodies had no significant impact on the safety, PK characteristics, or neutralizing activity of the SA55 injection. However, we also observed that the cumulative positive rate of ADA levels in the treatment group of the subjects was similar to that in the placebo group. First, the detection of ADA in placebo samples might be attributed to inherent characteristics of the immunogenicity assay. The determination method may cross-react with pre-existing antibodies, and these interactions may lead to false-positive signals (36). Second, there may be insufficient sample size. There is no large sample size available for formulating precise cut-off values. Therefore, using the commonly used cut-off values for analysis may lead to errors (37). Third, although our study excluded patients with diagnosed autoimmune disorders, subclinical immune activation or prior exposure to biologics (e.g., vaccines and infections) could elevate baseline antibody levels (38, 39).

In this study, a positive correlation was observed between the dose of SA55 injection and the blood concentration. The four dosage groups exhibited certain regularities in PK characteristics. Specifically, as the administered dose increased, T_max_ gradually shortened from approximately 12.6 days (150 mg group) to about 6 days (900 mg group). This indicates that the absorption rate of the drug in the body accelerates with increasing dosage. Concurrently, C_max_, AUC_0-t_, and AUC_0-∞_ all significantly increased with higher doses, displaying an approximately linear relationship. The t_1/2_ values for the 300, 600, and 900 mg groups were similar, ranging between 94 days to 103 days, showing no significant dose-dependent trend, suggesting that the elimination process of SA55 in the body is less influenced by the dose. Furthermore, with a half-life exceeding 3 months, the SA55 injection demonstrates potential for use in the prevention of COVID-19 infection. Its long half-life and rapid increase in concentration after use are conducive to rapid prevention and treatment of infected patients with severe risk factors.

Our study has several limitations that warrant consideration. First, as a phase I clinical trial, the investigation was constrained by a relatively small sample size. Consequently, all participants recruited for this study were healthy volunteers, which may not accurately reflect the PK profile in COVID-19-infected patients. These limitations underscore the necessity for further comprehensive research involving larger sample sizes to validate and extend our findings.

### Conclusion

The broad-spectrum neutralizing antibody SA55 injection against the novel coronavirus demonstrates good safety and tolerability in healthy populations. The neutralizing activity of the 600 mg dose group suggests it may be a potential target dose for further evaluation.

## Data Availability

Data sets generated during and/or analyzed during the current study are available from the corresponding author on reasonable request.
